# Therapeutic Approaches in Mitochondrial Dysfunction, Inflammation, and Autophagy in Uremic Cachexia: Role of Aerobic Exercise

**DOI:** 10.1155/2019/2789014

**Published:** 2019-08-18

**Authors:** Yumei Zhang, Yuqing Liu, Xiao Bi, Chun Hu, Feng Ding, Wei Ding

**Affiliations:** Division of Nephrology, Shanghai Ninth People's Hospital, School of Medicine, Shanghai Jiaotong University, 639 Zhizaoju Road, Shanghai 200011, China

## Abstract

Chronic kidney disease (CKD) causes several systemic changes, including muscular homeostasis, and eventually results in muscle atrophy. CKD-induced muscle atrophy is highly prevalent, and exercise is well known to enhance muscle function in these cases, although the exact mechanism remains unclear. Here, we aim to assess whether the protective effect of aerobic exercise in 5/6 nephrectomized (CKD) mice is associated with mitochondrial dysfunction, autophagy, or inflammation. C57BL/6J mice were randomly allocated into 3 different experimental groups: Sham, CKD, and CKD+aerobic exercise (CKD+AE). Renal function was assessed via serum creatinine and urea levels, and histological PAS and Masson staining were performed. Muscle wasting was determined based on grip strength, cross-sectional area (CSA), and MyHC protein expression. We also measured mitochondrial dysfunction in mice by assessing mtDNA, ROS, ATP production, and mitochondrial configuration. Autophagy was determined via assessments for Atg7, LC3, and SQSTM1 on western blotting. Inflammation was identified via proinflammatory cytokines and NLRP3 inflammasome components using real-time PCR and western blotting. We found that CKD mice exhibited higher BUN and creatinine levels and more severe glomerulosclerosis in the glomeruli and renal tubulointerstitial fibrosis, relative to the Sham group; all these effects were relieved by aerobic exercise. Moreover, grip strength, CSA, and MyHC protein expression were improved after 8 weeks of aerobic exercise. Furthermore, aerobic exercise significantly decreased MDA levels, increased SOD2 activity and ATP production, and improved mitochondrial configuration, relative to the CKD group. In addition, aerobic exercise downregulated the overexpression of proinflammatory cytokines and NLRP3 inflammasome components and balanced the mitochondrial biogenesis and autophagy-lysosomal system. Thus, we observed that aerobic exercise may ameliorate CKD-induced muscle wasting by improving mitochondrial dysfunction, inflammation, and autophagy-lysosomal system in uremic cachexia.

## 1. Introduction

Chronic kidney disease (CKD) is a complicated progressive disease that results in the progressive and irreversible loss of renal function and has become an increasingly important public health issue worldwide [1, 2]. CKD leads to a reduction in physical function during the initial stages, which gradually worsens as the disease progresses. Physical inactivity is primarily caused by skeletal muscle atrophy. A better understanding of the pathways that cause muscle wasting in CKD is vital to design appropriate therapeutic approaches to limit muscle protein loss. However, studies on the related mechanisms specific to CKD remain scarce. Thus far, several molecular mechanisms have been proposed to explain CKD-induced skeletal muscle atrophy, including oxidative stress injury or the upregulation of cyclic AMP and growth hormone/insulin-like growth factor 1 [3–5]. However, the exact mechanisms remain unclear. In the present study, we aim to determine the pathways causing muscle atrophy in this condition.

Exercise capacity is strongly related to mitochondrial function in the skeletal muscle. In particular, mitochondria are essential for maintaining skeletal muscle energy homeostasis, and muscle mitochondrial disruption is a novel pathophysiologic mechanism that contributes to the high prevalence of physical impairments in individuals with CKD [6, 7]. The amount of mitochondria is regulated by both mitochondrial biosynthesis and degradation. Exercise training is well known to enhance muscle mitochondrial function, leading to improvements in whole-body metabolic homeostasis. However, little information is available on the effects of aerobic exercise on skeletal muscle mitochondrial function and tissue homeostasis in individuals with CKD in vivo. Hence, further studies are needed to fully understand the mechanisms underlying mitochondrial biogenesis and quality that define muscle fitness.

Autophagy is a tightly regulated system wherein cellular protein aggregates and damaged organelles, including mitochondria, are removed via the lysosomal pathway [8]. A finely tuned regulation of autophagy flux appears to be responsible for maintaining muscle quality. Superfluous autophagy can lead to the excessive removal of cellular components such as mitochondria that are needed for normal activities, whereas insufficient autophagy can lead to the accumulation of damaged or dysfunctional cell components, which could result in muscle weakness. In addition to eliminating materials, autophagy also serves as a highly efficient recycling system that produces new components and energy for cellular renovation and homeostasis [9, 10]. Although autophagy is believed to play an important role in skeletal muscle remodeling, autophagy may also be upregulated under conditions of stress. Certain studies have shown that autophagy may be associated with muscle atrophy in several catabolic conditions [11–13], although the precise mechanism of autophagy in the muscle of CKD mice remains unclear.

Inflammation is a part of the normal response to tissue damage. However, overt or chronic inflammation can lead to secondary tissue damage and organ dysfunction. In particular, reduced kidney function leads to the retention of uremic solutes, which results in inflammation and oxidative stress and the impairment of skeletal muscle function [14]. Moreover, proinflammatory gene and protein expression are upregulated in the skeletal muscle of patients with CKD, leading to local and systemic inflammation and, consequently, to muscle atrophy [15]. In addition, inflammasome activation and subsequent release of proinflammatory cytokines are known to be involved in skeletal muscle wasting [16, 17]. NLRP3 is one of the four confirmed inflammasomes identified thus far. After formation and activation, the NLRP3 inflammasome associates with the adaptor protein ASC to activate caspase-1. Consequently, pro-IL-1*β* and pro-IL-18 are cleaved into their activated forms to trigger the downstream inflammation cascade [18]. The presence of the NLRP3 inflammasome has been determined not only in immunocompetent cells but also in cells responsible for various physiological functions, such as muscle cells. The NLRP3 inflammasome participates in age-related loss of muscle, and NLRP3 deletion mitigates the progression of sarcopenia in mice during aging [19]. Moreover, in a polymicrobial sepsis model, muscle atrophy was attenuated in septic Nlrp3 knockout mice, relative to septic wild-type mice [20]. Nevertheless, the function of the NLRP3 inflammasome in CKD-induced muscle wasting has not been completely elucidated. In the present study, we aimed to assess whether aerobic exercise can relieve muscle wasting by inhibiting the NLRP3 inflammasome in CKD mice.

In general, CKD is associated with impaired muscle mitochondrial metabolism. The skeletal muscle regulates the mitochondrial quality control mechanisms, as well as inflammation and autophagy, to maintain optimal muscle function. Limited but compelling evidence has shown that aerobic exercise can ameliorate muscle wasting and improve health quality in individuals with heart failure and uremia, although the potential underlying molecular mechanisms remain unclear. In the present study, we aim to assess whether the inhibition of muscle wasting in CKD mice via aerobic exercise is associated with mitochondrial dysfunction, autophagy, and inflammation and whether the specific training protocol used in this study can prevent muscle wasting.

## 2. Materials and Methods

### 2.1. Materials

Antibodies recognizing the following proteins were used in this study: caspase-3, Bax, LC3, ATG7, COXIV, *β*-actin, and HRP-conjugated secondary antibodies (Cell Signaling Technology, Beverly, MA); pro-IL-1*β*, IL-1*β*, PGC-1*α*, TFAM, and SQSTM1 (Abcam, Cambridge, MA); MyHC (R&D Systems, Minneapolis, MN); Nlrp3 and ASC (AdipoGen, San Diego, CA); pro-IL-18 and IL-18 (MBL, Minneapolis, MN); and procaspase-1 and caspase-1 (Santa Cruz Biotechnology, Santa Cruz, CA). The SOD2 assay kit, MDA assay kit, and ATP assay kit (Beyotime Biotechnology, China) were also used.

### 2.2. Animals and Groups

Male C57BL/6J mice (8–12 weeks old) were purchased from Shanghai Jestier Laboratory Animal Co. Ltd (Shanghai, China). All animal procedures were approved by the Institutional Animal Care and Use Committee of Shanghai Ninth People's Hospital, Shanghai Jiaotong University School of Medicine. All mice were housed with 12 h light/dark cycles. Mice were randomly divided into a control group (Sham; *n* = 6), a sedentary model group (CKD; *n* = 6), and an aerobic exercise model group (CKD+AE; *n* = 6). A CKD mouse model was established using 5/6 nephrectomy as previously described [21]. The Sham group underwent anesthesia induction and surgery without the removal of the kidney mass. At 2 weeks after subtotal nephrectomy, the CKD+AE group underwent adaptive training for another 2 weeks and then underwent formal aerobic exercise training for 8 weeks. When all the mice were killed, the kidney samples were collected for renal histopathology assessment and the gastrocnemius muscles were collected and placed in 10% paraformaldehyde or were immediately freeze-clamped and plunged into liquid nitrogen for subsequent RNA and protein analysis.

### 2.3. Aerobic Exercise Training Protocol

Two weeks before training, animals underwent a familiarization period on a wheel to become accustomed to the exercise protocol and handling. The familiarization running protocol involved an initial 5-minute warm up stage, and the speed was increased by 6 m/min, 3 times/one week. For aerobic exercise training, animals performed wheel running for 1 hour at a speed of 6.5 m/min, once a day, 5 times/week, for 8 weeks [22].

### 2.4. Renal Histopathology and Photomicrographs

Kidney and muscle samples fixed with 10% formalin were dehydrated in alcohol and cut into 4 mm slices. Periodic acid-Schiff (PAS) and Masson trichrome staining were performed following the protocol. At least 60 glomeruli were counted from 6 mice in each group, and the average glomerular injury score and tubulointerstitial fibrosis index were determined. All of these observations were made by 2 independent researchers who were blinded to the experimental groups.

### 2.5. Measurement of Hindlimb Grip Strength

The experimenter held the mice gently by the base of the tail, allowed the animals to grasp the transducer metal bar with their hindpaws, and pulled the animals away by their tail until grip was lost. To prevent mice from gripping the metal bar with their forepaws during the recording, the animals were first allowed to grasp a wire mesh cylinder with their forepaws. The peak force of each measurement was automatically recorded by the device. Hindlimb grip strength in each mouse was measured 3 times, and the average value was recorded.

### 2.6. Measurement of ROS and ATP Production

Skeletal muscle was solubilized in lysis buffer, and the lysate was centrifuged at 12,000 g for 5 minutes at 4°C to remove the insoluble portion. The protein content of supernatant fractions was quantified with the Enhanced BCA Protein Assay Kit (Beyotime, China). The concentration of malondialdehyde (MDA) was assayed using the thiobarbituric acid (TBA) method (S0131; Beyotime, China). SOD2 activity was determined by a Cu/Zn-SOD and Mn-SOD Assay Kit with WST-8 (S0103; Beyotime, China). ATP production was evaluated with the Enhanced ATP Assay Kit according to the manufacturer's instructions (S0027; Beyotime, China).

### 2.7. Electron Microscopy Assessment

Gastrocnemius muscles were fixed with 2.5% glutaraldehyde at room temperature and then cut into 1 mm^3^ pieces by using a scalpel. Ultrathin sections (60 nm) were prepared using a microtome and then placed on copper grids and stained with uranyl acetate and lead citrate for assessment via electron microscopy.

### 2.8. Western Blotting Analysis

Gastrocnemius muscles were homogenized in RIPA buffer (high) (Beyotime, China) with phosphatase inhibitor (Roche). Protein concentration was measured using an Enhanced BCA Protein Assay Kit. Equal amounts of protein were loaded on acrylamide/bis SDS-PAGE gels and transferred onto polyvinylidene fluoride (PVDF) membranes, which were blocked with 5% skim milk for 1 h. The PVDF membranes were incubated overnight at 4°C with the specific primary antibodies as follows: *β*-actin, MyHC, Bax, caspase-3, Atg7, LC3, SQSTM1, NLRP3, ASC, procaspase-1, caspase-1, pro-IL-1*β*, IL-1*β*, pro-IL-18, IL-18, PGC-1*α*, TFAM, and CoxIV. After washing with Tris-buffered saline Tween 20 (TBST), the membranes were incubated with HRP-conjugated secondary antibodies for 1 h. The results were analyzed using Quantity One software (Bio-Rad, Hercules, CA, USA).

### 2.9. Quantitative Real-Time Polymerase Chain Reaction

Total RNA were extracted and reverse-transcribed into cDNA following the instructions of the PrimeScript RT reagent kit (Takara, Dalian, Liaoning, China). Primer sequences for GAPDH, MyoD, myogenin, Pax-7, myostatin, atrogin-1, MuRF-1, IL-6, TNF-*α*, and mtDNA are listed in Table 1. RT-PCR analysis was performed with the 7500 Fast Real-Time PCR System (Applied Biosystems, Rockford, IL, USA). Melting curve analysis was always performed to analyze and verify the specificity of the reaction.

### 2.10. Statistical Analyses

Values are expressed as mean ± standard error of the mean (SEM). Differences between the 2 groups were compared using the *t*-test. When multiple treatments were compared, a one-way ANOVA was performed with a post hoc analysis with the Student-Newman-Keuls test. Differences were considered significant if the *P* values were <0.05.

## 3. Result

### 3.1. Improvement of Renal Function in CKD Mice via Aerobic Exercise

**Figure 1:**
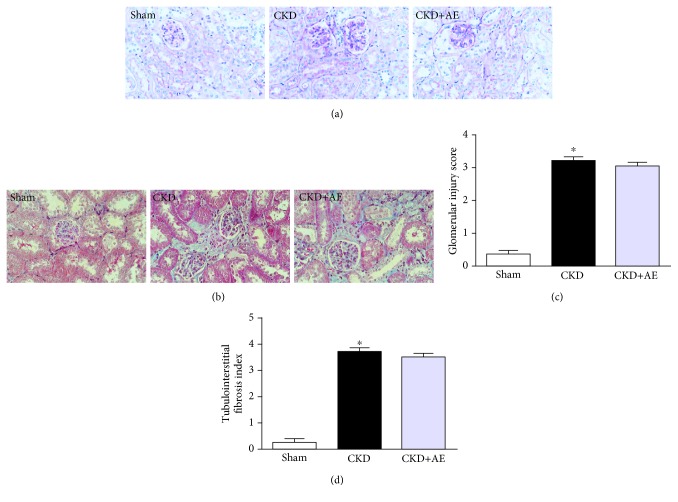
Aerobic exercise improved renal function in CKD mice. (a) Representative photomicrographs of periodic acid-Schiff-stained kidney sections (magnification, ×400). (b) Masson trichrome-stained kidney sections (magnification, ×400). (c) Glomerular injury scores and (d) tubulointerstitial fibrosis indices evaluated based on PAS and Masson staining. ^∗^*P* < 0.05, the CKD group vs. the Sham group. Data represent the mean ± SEM (*n* = 6).

CKD was induced via subtotal nephrectomy. The BUN and SCr levels in CKD mice exhibited an approximately 2-fold increase, relative to the Sham group (Table 2). Aerobic exercise treatment partly attenuated the increase in BUN and SCr levels in 5/6Nx mice. Periodic acid-Schiff (Figure 1(a)) and Masson-trichrome staining (Figure 1(b)) were used to observe the histopathological changes in the kidney. We found that the nephrectomy led to worsening of renal histopathology and the CKD group exhibited a greater renal injury score and tubulointerstitial fibrosis index. Aerobic exercise training slightly attenuated the worsening of renal histopathology, relative to the sedentary CKD group (Figures 1(c) and 1(d)), which suggests that aerobic exercise may have been able to partially counterbalance the deleterious effects following the 5/6Nx procedure.

### 3.2. Amelioration of CKD-Induced Muscle Wasting via Aerobic Exercise

**Figure 2:**
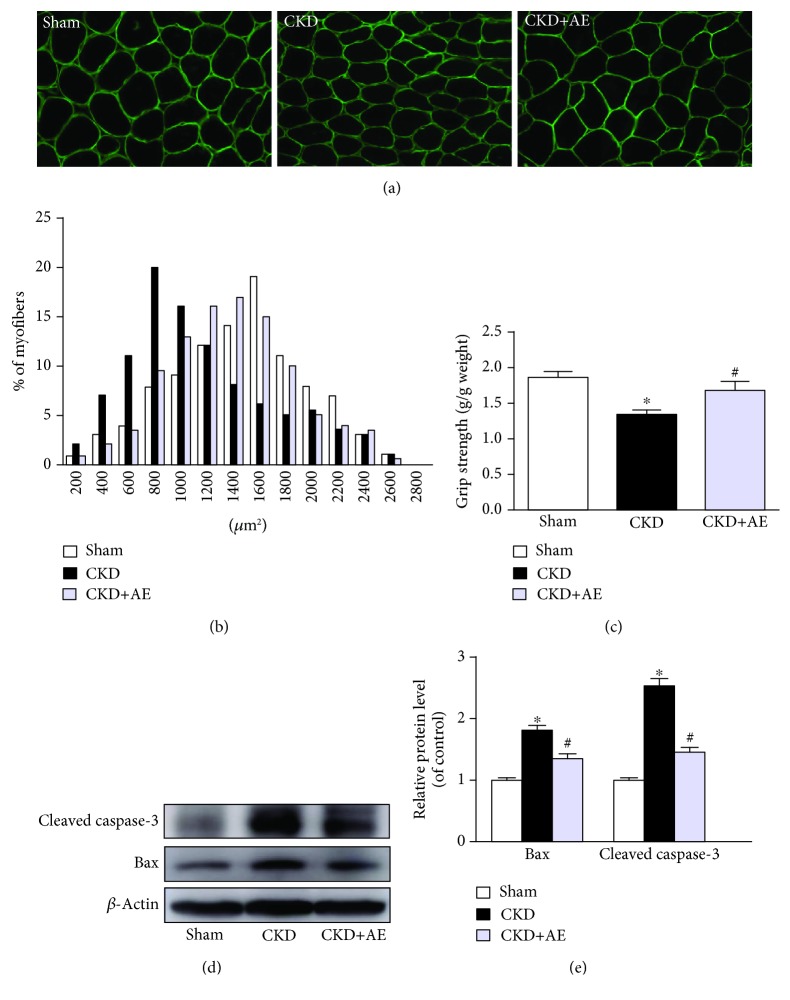
Aerobic exercise ameliorated CKD-induced muscle wasting. (a) Representative dystrophy staining images of cross-sectional areas (CSA) of the gastrocnemius muscle (magnification, ×400). (b) CSA distribution of the gastrocnemius muscle fiber. (c) Relative grip strength divided by body mass. (d) Western blot for Bax and cleaved caspase-3 protein expression in the Sham group and the CKD group with or without aerobic exercise training. (e) Quantification of Bax and cleaved caspase-3 levels, normalized against those of *β*-actin. ^∗^*P* < 0.05, the Sham group vs. the CKD group. ^#^*P* < 0.05, the CKD+AE group vs. the CKD group. Data represent the mean ± SEM (*n* = 6).

The body weight and gastrocnemius or tibialis anterior muscle dry weight were significantly lower in the CKD group, as compared to the Sham group. After 8 weeks of aerobic exercise training, the CKD mice exhibited markedly improved body weight and gastrocnemius muscle dry weight, although the effect on the tibialis anterior muscle was minimal (Table 2). Moreover, the CKD group showed a significant reduction in the cross-sectional area (CSA) of the gastrocnemius muscle (Figures 2(a) and 2(b)) and muscular strength (Figure 2(c)), relative to the Sham group. However, the CKD+AE group exhibited increased muscle strength and CSA, as compared to those in the CKD group. These data suggest a trend towards muscle atrophy in CKD and that aerobic exercise could help to overcome the deleterious effects of CKD on muscles. Western blotting analysis of the apoptosis markers demonstrated an increase in caspase-3 and Bax in CKD mice, relative to the Sham group. However, these levels decreased towards the baseline value after exercise training (Figures 2(d) and 2(e)).

### 3.3. Reversal of Muscle Protein Homeostasis Imbalance via Aerobic Exercise

**Figure 3:**
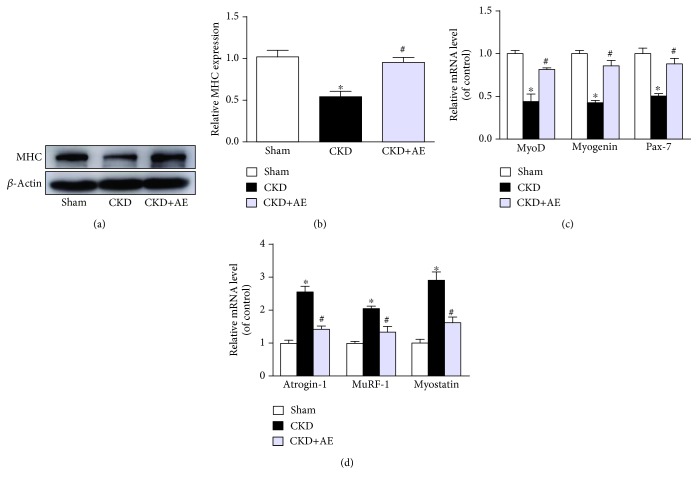
Aerobic exercise reversed the imbalance of muscle protein homeostasis. (a) Western blot of MyHC protein. (b) Quantification of MyHC levels, normalized against those of *β*-actin. (c) Changes related to markers of the dystrophin gene. (d) Gene expression of the markers of myogenesis, normalized against GAPDH performed by real-time PCR. ^∗^*P* < 0.05, the Sham group vs. the CKD group. ^#^*P* < 0.05, the CKD+AE group vs. the CKD group. Data represent the mean ± SEM (*n* = 6).

In the present study, the MyHC protein expression was downregulated in CKD mice and aerobic exercise reversed the CKD-induced MyHC protein reduction (Figures 3(a) and 3(b)). Moreover, the expressions of myogenic regulatory factors, such as MyoD, myogenin, and Pax-7, were decreased (Figure 3(c)), and the expressions of catabolic factors, such as atrogin-1, MuRF-1, and myostatin, were increased in the muscles of CKD mice, relative to the Sham group (Figure 3(d)). Aerobic exercise training blunted CKD muscle loss by decreasing muscle degradation and stimulating muscle protein synthesis.

### 3.4. Protection against Inflammation in CKD Mice via Aerobic Exercise

**Figure 4:**
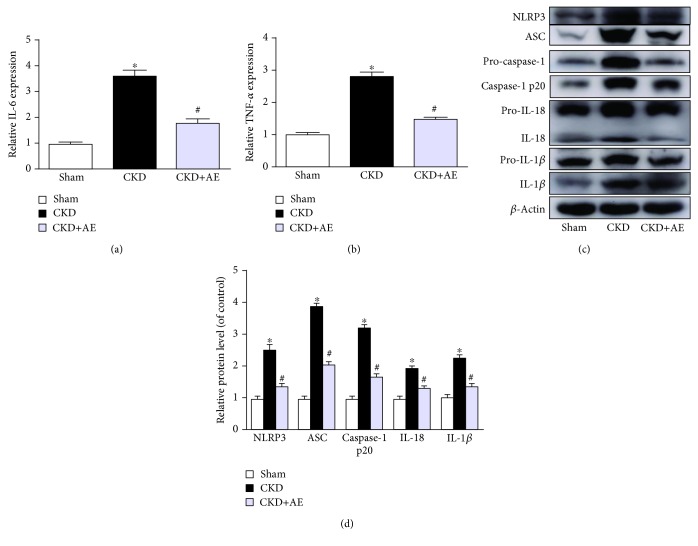
Aerobic exercise reduced muscle inflammation in CKD mice. (a) IL-6 and (b) TNF-*α* gene expression, normalized against GAPDH, assessed via real-time PCR. (c) Western blot of NLRP3, ASC, caspase-1, IL-18, and IL-1*β* expression. (d) Quantification analysis of NLRP3, ASC, caspase-1, IL-18, and IL-1*β* levels, normalized against *β*-actin. ^∗^*P* < 0 05, the Sham group vs. the CKD group. ^#^*P* < 0 05, the CKD+AE group vs. the CKD group. Data represent the mean ± SEM (*n* = 6).

Real-time PCR showed that nephrectomy leads to an increase in the gene expression of proinflammatory cytokines such as IL-6 (Figure 4(a)) and TNF-*α* (Figure 4(b)) and that aerobic exercise significantly decreased IL-6 and TNF-*α* levels. In addition, we found that CKD mice displayed a significant increase in the protein expression of NLRP3, ASC, caspase-1, IL-18, and IL-1*β*. However, the activation of the NLRP3 inflammasome and its downstream cytokines was significantly inhibited via treatment with aerobic exercise training (Figures 4(c) and 4(d)).

### 3.5. Attenuation of Muscle Mitochondrial Dysfunction in CKD Mice via Aerobic Exercise

**Figure 5:**
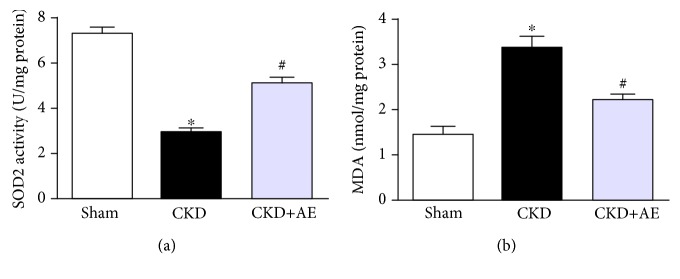
Aerobic exercise reduced muscle ROS production in CKD mice. (a) SOD2 activity and (b) MDA content measured after 8 weeks of aerobic exercise training. ^∗^*P* < 0.05, the Sham group vs. the CKD group. ^#^*P* < 0.05, the CKD+AE group vs. the CKD group. Data represent the mean ± SEM (*n* = 6).

**Figure 6:**
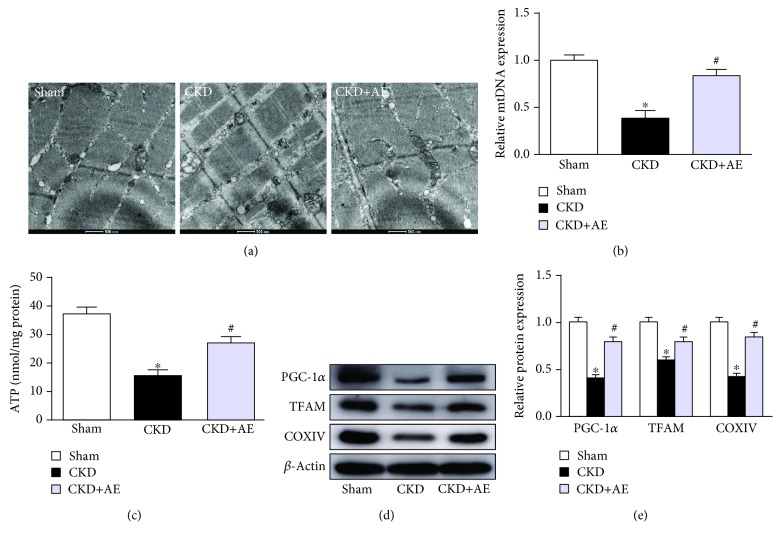
Aerobic exercise attenuated muscle mitochondrial dysfunction in CKD mice. (a) Morphological changes of mitochondria examined by transmission electronic micrographs (TEM) (magnification ×12,000). (b) Mitochondrial DNA (mtDNA) copy number, normalized against GAPDH, measured via real-time PCR. (c) ATP production, expressed as per mg protein. (d) Western bolt of PGC-1*α*, TFAM, and CoxIV expression. (e) Quantification analysis of PGC-1*α*, TFAM, and CoxIV levels, normalized against *β*-actin. ^∗^*P* < 0.05, the Sham group vs. the CKD group. ^#^*P* < 0.05, the CKD+AE group vs. the CKD group. Data represent the mean ± SEM (*n* = 6).

In the present study, mitochondrial SOD2 activity (Figure 5(a)) had declined and MDA content (Figure 5(b)) was elevated in CKD mice, although aerobic exercise markedly reduced oxidative stress. We observed morphological differences between the CKD group and aerobic exercise group on transmission electronic micrographs (TEM). The CKD group showed a greater abundance of swollen mitochondria with severely disrupted cristae. However, aerobic exercise enhanced mitochondrial activity, evidenced by the improved mitochondrial configuration (Figure 6(a)). The results of real-time PCR showed that the level of mitochondrial DNA (mtDNA), which is the primary target of free radicals, was lower in CKD mice, and this decrease was partially reversed via aerobic exercise (Figure 6(b)). ATP production was also decreased in the CKD group but was restored via aerobic exercise training (Figure 6(c)). Moreover, the levels of both peroxisome proliferator-activated receptor gamma coactivator-1*α* (PGC-1*α*) and mitochondrial transcription factor A (TFAM) were measured to evaluate mitochondrial biogenesis. Our data showed that the muscle protein levels of PGC-1*α* and TFAM were reduced in the CKD group and that these lower levels were reversed via aerobic exercise (Figures 6(d) and 6(e)). Moreover, we found that the critical mitochondrial protein CoxIV was markedly decreased in CKD mice and that aerobic exercise training partially ameliorated the damage (Figures 6(d) and 6(e)).

### 3.6. Inhibition of the Overactivation of Autophagy in CKD Mice via Aerobic Exercise

**Figure 7:**
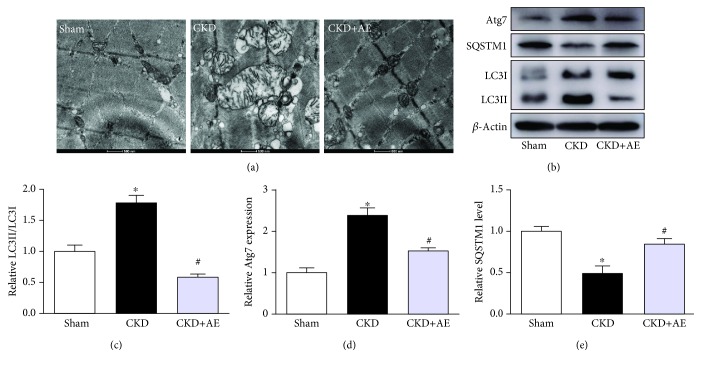
Aerobic exercise inhibited the overactivation of autophagy in CKD mice. (a) Images of morphology of autophagosomes with TEM (magnification, ×12,000). (b) Western blotting of autophagosome-related proteins LC3, Atg7, and SQSTM1. (c) Quantification of the ratio of LC3-II to LC3-I, normalized against that of *β*-actin. (d) Quantification analysis of the Atg7 level, normalized against that of *β*-actin. (e) Quantification analysis of the SQSTM1 content, normalized against that of *β*-actin. ^∗^*P* < 0.05, the Sham group vs. the CKD group. ^#^*P* < 0.05, the CKD+AE group vs. the CKD group. Data represent the mean ± SEM (*n* = 6).

As autophagy is critical for removing old or damaged organelles, including mitochondria, we investigated the ultrastructural phenotypes of autophagosomes in the 3 groups of mice under TEM, which is widely accepted as a suitable method for monitoring autophagy. Our results showed that CKD induced substantial autophagosome formation in skeletal muscle, relative to the Sham group (Figure 7(a)). The abundance of several key autophagy-related proteins, including LC3, Atg7, and SQSTM1, was observed in the skeletal muscle. We found that LC3 and Atg7 expressions were increased, whereas the SQSTM1 expression was reduced in the muscles of CKD mice, in comparison to Sham-operated animals. This finding suggests that autophagy was induced in the skeletal muscle of CKD mice (Figures 7(b)–7(e)). However, the increased LC3 and Atg7 protein levels and decreased SQSTM1 protein levels in the muscles of CKD mice were significantly reversed via treatment with aerobic exercise. These results indicate that aerobic exercise inhibited the overactivation of autophagy in skeletal muscles.

## 4. Discussion

Various aerobic exercise protocols have been administered to CKD patients to prevent the atrophy of skeletal muscles and have found to offer physiological benefits for this patient population [23]. However, the molecular mechanisms underlying skeletal muscle dysfunction and the response to aerobic exercise remain unclear. In the present study, we found that factors such as mitochondrial dysfunction, inflammation, and autophagy played a very critical role in CKD-induced muscle wasting. Aerobic exercise may attenuate the loss of muscle mass in CKD mice by enhancing grip strength and muscle CSA through the regulation of mitochondrial dysfunction, inflammation, and autophagy. Thus, aerobic exercise may represent a viable treatment for CKD-induced muscle wasting.

Muscular-motor protein breakdown is a prominent feature of muscle atrophy [24]. In the present study, we found that a reduction in the MyHC protein expression was accompanied by muscle atrophy in CKD mice, relative to Sham-operated mice. Previous studies have found that muscle wasting is caused by the activation of atrophy-related gene transcription in addition to the expression of the myogenic factors [25]. Our data show that CKD can activate the expression of atrophy-related genes such as atrogin-1, MuRF1, and myostatin and can also decrease the expression of myogenic regulatory factors such as MyoD and myogenin. However, aerobic exercise reversed this trend. We suggested that aerobic exercise training inhibits CKD-induced muscle loss by decreasing muscle proteolysis and stimulating muscle protein synthesis.

Mitochondria are known to be essential for maintaining skeletal muscle energy homeostasis, and the deterioration of mitochondrial function, including decreased ATP output, increased MDA, and decreased SOD2 production, is often observed under uremic conditions [26]. Skeletal muscle protein turnover, particularly that of mitochondrial proteins, plays a central role in mediating the health benefits of aerobic exercise training. Mitochondrial biogenesis requires a coordinated action of nuclear and mitochondrial genomes that are regulated by the coactivators PGC-1*α* and TFAM. One major finding of this study is that mtDNA and ATP levels were reduced in CKD mice in parallel with incremental ROS production, and these effects were partially reversed by aerobic exercise. Another major finding is that the PGC-1*α* and TFAM expression was increased in skeletal muscle following aerobic exercise, relative to the CKD group. These results are consistent with our TEM discovery, which indicated that CKD mice exhibited greater amounts of swollen mitochondria with severely disrupted cristae, whereas aerobic exercise reversed this trend. These results also show that mitochondrial dysfunction may be a key target through which aerobic exercise ameliorates CKD-induced muscle wasting.

CKD and related inflammatory responses stimulate protein-energy wasting, causing the loss of muscle mass. Previous studies have found that the expression of traditional proinflammatory cytokines such as IL-6 and TNF-*α* is greater in muscle wasting. We also observed an upregulation of IL-6 and TNF-*α* in CKD mice and a significant decrease in these levels following aerobic exercise. [27–29]. Recently, Yet and coworkers showed that the neutralization of IL-6 and TNF-*α* could not entirely restore inflammation homeostasis and that there might be other inflammatory cytokines involved. The NLRP3 inflammasome is one such research hotspot in inflammation and is reportedly responsible for various physiological functions. A few studies have indicated that the NLRP3 inflammasome plays a role in muscle wasting [30, 31]. Our study also found that NLRP3 inflammasome activation was increased and the expression levels of its downstream cytokines, including caspase-1, IL-18, and IL-1*β*, were greater in the muscles of the CKD group; nevertheless, aerobic exercise markedly attenuated NLRP3 inflammasome activation. These results offer further support for the theory that aerobic exercise may ameliorate muscle wasting by inhibiting inflammatory reactions, including those induced by the NLRP3 inflammasome.

An appropriate balance between mitochondrial biogenesis and removal via autophagy is essential for healthy mitochondria and skeletal muscle. Previous studies showed that autophagy was involved in the progression of skeletal muscle wasting in CKD [32]. A certain extent of autophagy is beneficial to muscle health, but the persistent activation of autophagy accelerates disease progression [33]. In the present study, LC3 and Atg7 expressions were increased, whereas the SQSTM1 protein expression was reduced in the gastrocnemius muscle of the CKD group, relative to the Sham group. However, aerobic exercise training prevented excessive autophagy in the muscles of the CKD group. Overall, autophagy was markedly increased in CKD mice but was also greatly inhibited by treatment with aerobic exercise. Thus, aerobic exercise inhibits CKD-induced excessive autophagy, which may represent an adaptive response to protect against injury.

In conclusion, in the present study, we found that mitochondrial dysfunction, NLRP3 inflammasome, and autophagy were involved in CKD-induced muscle wasting and that aerobic exercise training could reverse muscle atrophy in uremic cachexia by ameliorating mitochondrial function and relieving autophagy and inflammation. The present study offers evidence of a novel downstream pathway in CKD-induced skeletal muscle wasting and suggests that aerobic exercise training might represent an appropriate therapeutic approach to muscle wasting in CKD patients.

## Figures and Tables

**Table 1:** Gene sequences used in this study.

Gene	Forward primer sequence (5′–3′)	Reverse primer sequence (5′–3′)
mtDNA	TTTTATCTGCATCTGAGTTTAATCCTGT	CCACTTCATCTTACCATTTATTATCGC
IL-6	TAGTCCTTCCTACCCCAATTTCC	TTGGTCCTTAGCCACTCCTTC
TNF-*α*	CCCTCACACTCAGATCATCTTCT	GCTACGACGTGGGCTACAG
MyoD	CCACTCCGGGACATAGACTTG	AAAAGCGCAGGTCTGGTGAG
Myogenin	GAGACATCCCCCTATTTCTACCA	GCTCAGTCCGCTCATAGCC
Pax-7	TCTCCAAGATTCTGTGCCGAT	CGGGGTTCTCTCTCTTATACTCC
Atrogin-1	CAGCTTCGTGAGCGACCTC	GGCAGTCGAGAAGTCCAGTC
MuRF-1	GTGTGAGGTGCCTACTTGCTC	GCTCAGTCTTCTGTCCTTGG
Myostatin	AGTGGATCTAAATGAGGGCAG	GTTTCCAGGCGCAGCTTAC
GAPDH	AGGTCGGTGTGAACGGATTTG	TGTAGACCATGTAGTTGAGGTCA

**Table 2:** Renal function, body weight, and muscle weight profile for the Sham, 5/6-nephrectomized (CKD), and aerobic exercise training (CKD+AE) mice at week 12.

	Sham	CKD	CKD+AE
SCr (mg/dl)	0.15 ± 0.03	0.32±0.02^∗∗^	0.29 ± 0.01
BUN (mg/dl)	25.0 ± 3.0	58.6±2.9^∗∗^	55.1 ± 3.1
Final body weight (g)	29.0 ± 0.7	23.2±0.9^∗∗^	27.4 ± 0.5^##^
Gastrocnemius weight (mg)	158.1 ± 8.2	128.2±7.4^∗∗^	148.4 ± 9.8^##^
Tibialis anterior weight (mg)	58.2 ± 4.7	52.5 ± 3.1^∗^	56.5 ± 1.4

Data are presented as mean ± SD. ^∗^*P* < 0.05, ^∗∗^*P* < 0.01, the Sham group vs. the CKD group. ^#^*P* < 0.05, ^##^*P* < 0.01, the CKD group vs. the CKD+AE group. CKD: chronic kidney disease; BUN: blood urea nitrogen; SCr: serum creatinine.

## Data Availability

The data used to support the findings of this study are available from the corresponding authors upon request.
